# Modeling biological age and its link with the aging process

**DOI:** 10.1093/pnasnexus/pgac135

**Published:** 2022-07-26

**Authors:** Hiram Beltrán-Sánchez, Alberto Palloni, Yiyue Huangfu, Mary C McEniry

**Affiliations:** Fielding School of Public Health and California Center for Population Research, UCLA, Los Angeles, CA 90095, USA; Center for Demography of Health and Aging, University of Wisconsin–Madison, Madison, WI 53706, USA; Consejo Superior de Investigaciones Cientificas, CSIC, Madrid 28006, Spain; Center for Demography of Health and Aging, University of Wisconsin–Madison, Madison, WI 53706, USA; Center for Demography of Health and Aging, University of Wisconsin–Madison, Madison, WI 53706, USA

**Keywords:** biological age, aging, mortality, biomarkers

## Abstract

Differences in health status at older ages are a result of genetic predispositions and physiological responses to exposure accumulation over the lifespan. These vary across individuals and lead to health status heterogeneity as people age. Chronological age (CA) is a standard indicator that reflects overall risks of morbidity and mortality. However, CA is only a crude proxy for individuals’ latent physiological deterioration. An alternative to CA is biological age (BA), an indicator of accumulated age-related biological change reflected in markers of major physiological systems. We propose and validate two BA estimators that improve upon existing ones. These estimators (i) are based on a structural equation model (SEM) that represents the relation between BA and CA, (ii) circumvent the need to impose arbitrary assumptions about the relation between CA and BA, and (iii) provide tools to empirically test the validity of assumptions the researcher may wish to invoke. We use the US National Health and Nutrition Examination Survey 1988–1994 and compare results with three commonly used methods to compute BA (principal components—PCA, multiple regression—MLR, and Klemera–Doubal’s method—KD). We show that SEM-based estimates of BA differ significantly from those generated by PCA and MLR and are comparable to, but have better predictive power than KD’s. The proposed estimators are flexible, allow testing of assumptions about functional forms relating BA and CA, and admit a rich interpretation as indicators of accelerated aging.

Significance Statement Several methods have been proposed to estimate biological age (BA) from available biomarkers, all of them rely on linear regression approaches, and all constrain the functional form of the relation between chronological age (CA) and BA. Structural equation model (SEM)-based estimators relax assumptions about functional forms and do not impose parameter constraints. Our empirical results suggest that parameter constraints invoked by other methods are unnecessary and could produce misleading inferences. One of the proposed BA estimators, outcome-dependent, is the only one that suggests faster underlying physiological deterioration relative to CA. This is consistent with accelerated biological aging and senescent mortality. SEM-based estimators could provide empirical evidence to discriminate between competing mechanisms proposed in evolutionary biology to explain senescence and mortality acceleration.

## Introduction

Differences in health status at older ages are the result of biological processes responsive to accumulation of exposures across the lifespan. These exposures are highly diverse, their timing stretches from preconception and in-utero environments to adulthood, vary widely across individuals, and lead to sizeable heterogeneity in the pace of health status changes experienced as people age. Chronological age (CA) is a standard, easy to measure, indicator of health changes because it is correlated with observable markers such as the incidence of chronic disease, disability, and death. However, CA is a crude proxy of the underlying physiological processes that induce deterioration. An alternative to CA is biological age, BA, an indicator of age-related latent physiological change computed with the aid of a battery of biomarkers of major physiological domains (1–5). Estimates of BA are more strongly associated than CA with many physical health outcomes (3, 6) and also depressive symptoms (7).

Methods currently in use to estimate BA rely on regression approaches in which BA is modeled as a linear function of CA and an array of biomarkers. We use the term *biological markers* to refer to general indicators of functioning of multiple systems [including DNA (base pairs sequence), DNA protein production (transcription, translation), metabolites, and chromatin state and markers of physiological domains (metabolic, respiratory, cardiovascular)]. We reserve the term *biomarkers* to refer to a subset of biological markers known as indicators of physiological states. These include plasma proteins, urine metabolites, and external markers such as body temperature, blood pressure, pulse, BMI, and waist circumference. When no confusion is likely, we will simply use the shortcut “marker”. Empirical evidence suggests that the predictive power of a method proposed by Klemera and Doubal (2) is superior to that of two traditional methods (principal components—PCA and multiple regression—MLR) (3). In this paper, we propose two flexible estimators that improve empirical performance and admit interpretations as indicators of latent physiological deterioration. The first estimator is “outcome-free” and depends solely on the parameters of a structural equation model (SEM) that represents relations between a latent construct, BA, observed CA, and observable biomarkers. The second estimator is “outcome-dependent (OD),” also requires SEM and, in addition, must be anchored on the modeling of an outcome of physiological aging. Although the current version of this method we discuss here uses mortality, other health outcomes are also plausible anchors.

The next section briefly reviews the concept of biological aging and the utility of BA. The “Materials and methods” section describes existing BA estimators, introduces two new ones, and applies the proposed estimators to the prediction of adult mortality. The “Data” section summarizes the National Health and Nutrition Examination Survey (NHANES) 1988–1994 data set and identifies the biomarkers used in the analysis. In the “Results” section, we review estimation strategies, compare BA estimates from different methods, and examine the behavior of the difference between BA and CA, $\Delta BA_{t_0}$, as a predictor of adult mortality. The “Summary and discussion” section summarizes, identifies limitations, and proposes extensions.

## BA and senescence: Is BA a useful concept?

Progressive physiological deterioration is of growing interest in biomedical and aging research, and has been studied using an indicator referred to as BA (2, 4, 8). An ideal estimator of BA should be based on joint information about multiple domains ranging from standard biomarkers of selected physiological states such as blood pressure, glycated hemoglobin, lipid profiles (2), to genetic markers such as telomere length, to indicators of neurodegenerative activity such as plasma NfL, total-tau, and amyloid beta-40 and -42 (9), to markers of epigenetic modifications, including DNA’s differentially methylated sites (10). However, and up until recently, the indicators available to population health researchers in large, nationally representative surveys are limited to one or at most two domains, physiological states, and (epi)genetics [more indicators are available in small epidemiologic and cohort studies (11, 12)].

Existing methods to estimate BA are based on indicators for either epigenetic or physiological functioning. For example, epigenetic clocks, a surrogate of BA, do not require information on biomarkers (10) and, conversely, estimators of BA based on biomarkers include neither epigenetic nor genetic information. There is some empirical evidence suggesting that BA estimates based on a limited number of readily available biomarkers are good predictors of mortality (3,6), onset of chronic illnesses, including stroke and cancer (9), and even cognitive and physical decline (11). In theory, and because it must reflect latent physiological status, BA should also be sensitive to distal exogenous stressors, such as socioeconomic and environmental factors, or to proximate conditions, such as medical interventions and health behavior modifications. Finally, BA will also be affected by the inherent stochasticity of the aging process (13,14).

Thus defined, BA is a latent variable that need not march in lockstep with CA within finite life span segments. Thus, for example, as a result of successful treatment of chronic conditions, physiological functioning may improve, albeit transiently, and thereby decelerate biological aging even though CA maintains its pace of increase. In this case, and only in the range of ages within which the interventions have effects, there will be a weak association between BA and CA. This may have happened in some subpopulations after adoption of interventions to lower cholesterol and blood pressure (15). The reverse is also possible: Individuals who are exposed to teratogenes early in life may experience excess deterioration at adult ages and BA will increase more rapidly than CA after individuals attain critical ages beyond which the delayed effects of early exposures is manifested. Similarly, adoption of deleterious health behaviors, such as smoking, poor diet, alcohol consumption, and lack of physical activity, could accelerate aging above and beyond what is expected from the passage of CA(An example of apparent disharmony between BA and CA is findings showing that while the US population is aging (i.e. average CA is increasing), BA is declining (6, 11)).

Finally, BA may prove useful in two different areas of research. First, it could provide empirical evidence to discriminate between competing mechanisms invoked in evolutionary biology to account for senescence and mortality acceleration (14, 16–20). Furthermore, it will enrich the demographic tool kit to study population aging and broaden available empirical evidence, which is currently limited to the workhorse in the area, e.g. country-based aggregate mortality rates (21–23).

## Results

### Summary statistics

Descriptive statistics for the biomarkers included in the NHANES 1988–1994 sample for the total population and for men and women are shown in Appendix Table S1. About half (52.6%) of the participants are women with an average age of 49.2 y. Men are slightly older, with a mean of 50.3 y, and had higher average values than women in most of the physiological systems, including indicators of cardiac, lung, kidney, and liver function. In contrast, there were no important gender differences in markers of metabolic and immune/inflammation. With two exceptions (albumin and forced expiratory volume), higher values in a biomarker are associated with worse underlying physiological status(The exceptions are consistent with the underlying physiological meaning of these biomarkers: Lower values in FEV correspond to worse lung functioning while low albumin values are related to heart attack, stroke, functioning loss, and death among older adults (24)).

Tables in the Appendix (Tables S2 to S5) display parameter estimates of the linear and nonlinear SEM models for the total, female, and male samples. According to all three measures of fit (Appendix Table S5), the linear form of the SEM model performs marginally better than the log linear form in all three samples. In both model specifications, the estimated coefficients of effects of BA on biomarkers are statistically significant, contained within CIs excluding zero, larger for males than for females, and, finally, other than those for expiratory volume (FEV) and albumin, positively associated with BA (see above).

### Estimates of BA

We first compute BA using SEM model parameters (Appendix Sections SI and SII). Each individual in the sample is assigned four BA values: Two (linear and nonlinear SEM) associated with the outcome-free method (OF) and two (linear and nonlinear SEM) associated with the OD estimate. Other estimates of BA are computed according to each method’s protocol. To test hypotheses about the relations between BA and CA, we estimate the linear association between BA and CA, on one hand, and between CA and the difference between BA and CA ($\Delta BA_{t_0}$), on the other. Results are shown Appendix Table S6. As should be expected from the method’s assumptions, Klemera–Doubal’s method (KD) produces an intercept equal to 0 and slope equal to 1 for the linear association between BA and CA for both females and males. In contrast, all other methods imply intercepts and slopes that are statistically different from 0 and 1, respectively, in violation of KD assumptions. Estimates of the slope of BA on CA in the MLR, PCA, and the OF SEM are smaller than 1. The OD SEM is the only method generating an estimated slope greater than 1. For example, among males, the estimated slopes attain values 0.48 (MLR), 0.62 (PCA), 0.87 (linear and nonlinear OF), and 1.20 (linear and nonlinear OD). Because the OD method is the only one that uses the outcome mortality to define BA, the slope of BA on CA will reflect the impact of both age related physiological deterioration assessed by biomarkers as well as the progression of mortality risks with CA driven by factors unrelated to the biomarkers. The other methods, particularly MLR and PCA, suggest *decreasing* physiological deterioration with CA. This is a pattern that could be explained by mortality selection. If so, it suggest that the OD method is less sensitive to selection than alternative methods.

Finally, the distributions of BA estimates are different across methods. Those computed from either PCA or MLR have larger variances and are more symmetrical and normal-like than those from other methods (Appendix Figure S2). Estimates from KD and the linear and nonlinear variants of the OF method, produce bi-modal age-patterns, with values concentrated between ages 30 and 40 and 60 to 70. Estimates from the two variants of the OD method are similarly bi-modal but with later peaks in the age range 70 to 80.

### Peculiarities of the OD method

Estimates from the SEM OD method are trained on observed mortality and have special properties. They depend strongly on the (mortality) predictive power of the SEM latent variable reflected in SEM’s factor scores. By construction (see Appendix Sections SI to SIII), when the effect of factor scores on mortality is zero, the OD estimate of BA will be identical to CA. Conversely, nonzero effects will translate into values of BA that depart from CA. None of the other methods requires this property to identify estimates of BA different from CA, since their values are unrelated to the predictive power for any outcome we care to choose. But therein lies the strength of the OD method: If the researcher’s final goal is to predict an outcome using the mapping of biomarkers on BA, it is best to use an OD method trained on such outcome. The procedure will produce both more accurate predictions and BA’s estimates with a richer interpretation.

The OD method proposed here relies on information about the predictive power of biomarkers (as reflected in SEM factor scores) in relation to a well-defined health outcome(In this paper, we use mortality, but other outcomes such as a chronic condition or even disability could be a legitimate target). Instead, OF methods only rely on the power of biomarkers to track chronological age. As a consequence, comparing the predictive power of an OD and an OF method is uninformative, unless the predicted outcome is not the same on which the OD method relies. The claim we are making is not about superiority of the OD over OF methods. Rather, we argue that metrics of BA that are outcome-specific are more appealing because alternative health outcomes are differentially sensitive to subsets of biomarkers. Physiological aging involves multiple systems and not all available biomarkers are equally responsive to them. As a consequence, a measure of BA that is outcome-specific is more meaningful and more interpretable than one that relies on the relation between CA and biomarkers but ignores the physiological manifestation of deterioration. If, for example, the set of biomarkers in the study happens to be biased toward those associated with metabolic function, it would be more meaningful to use an indicator of BA that depends on an external manifestation of metabolic dysfunction, say Type II Diabetes. Some health outcomes may be strongly related with each other and, in such cases, the various OD measures of biological age the researcher could formulate will be related. If so, it would be possible to predict one health outcome using an OD indicator of BA based on a different health outcome.

To compute the OD estimate, we first estimate two Gompertz mortality models: The first is a null model that uses an individual’s duration under observation in NHANES (since baseline survey) and a single control for CA at baseline. With appropriate constraints, the estimated coefficient of duration corresponds to a standard Gompertz slope, e.g. reflects the rate of increase of mortality rates with the passage of time. We interpret this as a measure of the speed of senescence or aging. In a second, augmented model, we also include SEM factor scores. In doing so, we are effectively controlling for variables representing physiological deterioration that were omitted in the null model. If these factor scores truly reflect what we think they do, the magnitude of the estimate of the duration slope in the augmented model should be lower than in the null model.

Table 1 displays estimates from the SEM linear model. It includes the null Gompertz hazard model controlling for CA at baseline, $CA_{t_0}$, and, in addition, an augmented Gompertz model that includes a control for SEM’s raw factor scores. Accelerated aging can be gauged by both the Gompertz slope and, equivalently, by the mortality rate doubling time associated with each Gompertz slope (25). Among women, the reduction in slope is of the order of 30% whereas the increase in the doubling time from 8 to about 11.3 is 42%. This means that physiological deterioration embedded in factors scores, account for an extra 42% in BA acceleration (similar results hold for males). If the set of biomarkers had been more extensive, the reductions should have been larger. In the limit, if we could account for all relevant biomarkers as well as other indicators of systems’ failure, the Gompertz slope in the augmented model should converge to 0 and only the level parameters would be relevant.

**Table 1.** Parameters of hazard models for the SEM linear and OD estimator.

			**SEM: linear**	
	**CA**	**95% CI**	**CA and FS(BA)**	**95% CI**
Females				
	0.087 (8.0)	[0.08, 0.09]	0.061 (11.3)	[0.06, 0.07]
*k*	5 × 10^−5^	[3 × 10^−5^, 6 × 10^−5^]	4 × 10^−5^	[3 × 10^−5^, 5 × 10^−5^]
FS(BA)	—	—	1.517	[1.26, 1.77]
Males				
	0.080 (8.7)	[0.08, 0.08]	0.056 (12.4)	[0.05, 0.06]
*k*	1.1 × 10^−4^	[9 ×10^−5^, 1.5 × 10^−4^]	1 × 10^−4^	[7 × 10^−5^, 1.3 × 10^−4^]
FS(BA)	—	—	1.837	[1.54, 2.13]

FS(BA) = raw factors scores from SEM model. Column CA shows estimates of *β* from a null Gompertz model: $\mu (CA(t))=k \cdot \exp (\beta \cdot CA_{t_0})\cdot \exp (\beta \cdot t)$. The column CA and FS(BA) shows estimates from an augmented Gompertz model that also includes FS(BA), namely, $\mu (CA(t))=k \cdot \exp (\beta ^{\prime } \cdot CA_{t_0}) \cdot \exp (\beta ^{\prime } \cdot t) \cdot \exp (\gamma \cdot FS(BA))$. Values in parentheses in columns CA and CA and FS(BA) are the doubling times associated with slopes (see Appendix Table S7 for SEM nonlinear).

### Predicting mortality

Table 2 shows estimated effects on mortality of the *difference between CA and BA*, $\Delta BA_{t_0}$. We use Gompertz survival models for the risk of death and model the event as a function of duration under observation, $CA_{t_0}$, and the difference BA–CA derived from each of the methods under study. First, the fit of the SEM models is best, particularly the OD variant, which is clearly superior in Akaike Information Criterion (AIC) and Bayesian Information Criterion (BIC) metrics. Second, estimated effects of the difference $\Delta BA_{t_0}$ ranges from a low of 0.031 (PCA) to a high of 0.254 (OD, linear SEM). We interpret these coefficients as the magnitude of the impact on mortality of cumulative, latent physiological damage not captured by $CA_{t_0}$. The two variants of the OD SEM method yield estimates that are as large as those from other methods. Thus, while estimated effects in the linear variant of the OF method for females translates into increases of 10% [100*exp(.097)] per unit of difference between BA and CA at baseline, the excess mortality rate in the OD method is approximately 1.28 = [100*exp(.254)]. The two SEM linear variants produce estimates similar in magnitude to those from the KD method but are twice as large as those from MLR and PCA. Finally, note that in all cases, the estimated Gompertz slope for males and females are within the narrow range expected for human mortality, 0.07 to 0.12, but the lowest values are associated with the OD method (13, 14, 17).

**Table 2.** Coefficient estimates from Gompertz proportional hazard models using the difference between BA and CA, $\Delta BA_{t_0}$, as a predictor of the risk of death (NHANES 1988–1994 and 2015 mortality follow-up).

**Parameter**	**KD**	**MLR**	**PCA**	**SEM: linear**		**SEM: nonlinear**	
				**OF**	**OD**	**OF**	**OD**
Female							
$\Delta BA_{t_0}$	0.072***	0.043***	0.031***	0.097***	0.254***	0.071***	0.243***
*β*	0.091***	0.105***	0.098***	0.102***	0.072***	0.097***	0.074***
*k*	0.00003	0.00001	0.00002	0.00002	0.00019	0.00002	0.00016
AIC	13,696.8	13,819.1	13,821.2	13,666.0	12,551.8	13,670.8	12,620.7
BIC	13,716.2	13,838.5	13,840.6	13,685.5	12,571.2	13,690.3	12,640.2
Males							
$\Delta BA_{t_0}$	0.061***	0.041***	0.030***	0.083***	0.223***	0.061***	0.215***
*β*	0.083***	0.100***	0.092***	0.093***	0.065***	0.089***	0.067***
*k*	0.00009	0.00003	0.00005	0.00005	0.00043	0.00006	0.00037
AIC	15,733.0	15,839.3	15,819.7	15,710.6	14,478.4	15,726.1	14,546.5
BIC	15,752.1	15,858.4	15,838.8	15,729.7	14,497.6	15,745.2	14,565.6

***P-value < 0.000. Parameter estimates correspond to the model: $\mu (CA(t))=k \cdot \exp (\beta \cdot CA_{t_0})\cdot \exp (\phi \cdot \Delta BA_{t_0})\cdot \exp (\beta \cdot t)$. Since we constrain the coefficient of $CA_{t_0}$ to be identical to the coefficient of duration, its value is the same as *β* in the table.

To compare the predictive power across models, we estimate the area under the receiver operating characteristics curve (AUC) (see Appendix Table S8). Higher AUC values are typically associated with better predictive performance. The table shows estimates for three levels of specificity (80% to 90%, 90% to 100%, and 0% to 100%). These results confirm that the SEM-based methods perform better. For example, the OD method (both linear and nonlinear) has the highest AUC value across all specificity levels and attains an overall predictive power (0% to 100%) of over 90% for both women and men.

**Fig. 1:**
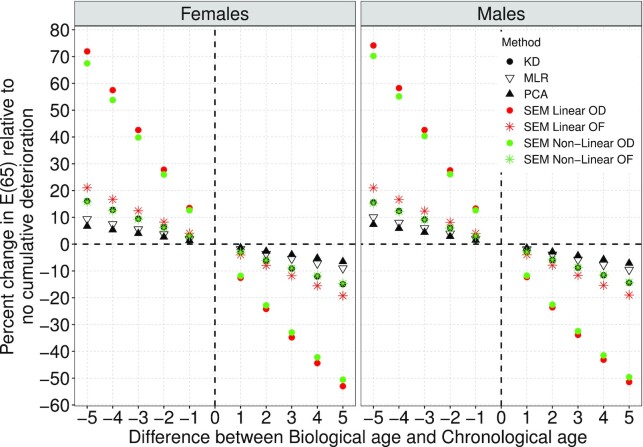
Relative changes in life expectancy at age 65, E(65), for a given change in BA relative to that of individuals with no cumulative latent deterioration at baseline $BA_{t_0}=CA_{t_0}$ estimated from Gompertz models from Table 2.

To better assess the implications of estimates in Table 2, we resort to a more easily interpreted metric, namely, life expectancy at age 65, E(65). We compute predicted values of E(65) using the estimated hazard model in alternative scenarios for BA and CA. To define these scenarios, we vary the difference $\Delta BA_{t_0}$ in the range between 0 and 5 y, i.e. a typical individual would be between 0 and 5 y biologically younger or older than her CA. We contrast predicted values of E(65) for individuals whose BA differs from their CA at baseline with those with $BA_{t_0}=CA_{t_0}$. Figure 1 displays values for the corresponding differences. The figure includes four quadrants, each associated with a combination of difference between BA and CA (positive or negative) and difference in predicted E(65) (positive or negative). Two regularities stand out. First, as expected, all points fall in the upper left and lower right quadrants, e.g. increases (decreases) in the differences between BA and CA lead to declining (increasing) E(65). Second, the rate of decline of E(65) from the OF and OD are larger than in other cases. In particular, the PC and MLR estimates lead to changes in E(65) that are shallower and less sensitive to variation of the indicator of physiological deterioration.

### Application: Accelerated aging by gender, race/ethnicity, and education

What is the relation between $\Delta BA_{t_0}$ and individual characteristics? To study this, we estimate models for $\Delta BA_{t_0}$ that include dummy variables for gender, race/ethnicity, and educational attainment. We use education and race/ethnicity since, in the United States at least, these are the two most important individual characteristics associated with poor health and mortality. The race and ethnicity categories correspond to standard groups employed in population health literature. The goal is to determine whether OD estimates lead to inferences about disparities that are consistent with that depend on more conventional outcomes (self-reported health, morbidity, and mortality). Table 3 displays results. According to AIC and BIC metrics, the SEM methods have a better overall fit. Also note that, as forced by initial constraints, the KD’s estimates are close to zero. With the exception of the nonlinear SEM, age effects are negative and statistically significant. Furthermore, the estimated effects of educational attainment and race/ethnicity are as expected: Non-Hispanic Blacks and Mexican American have positive differences $\Delta BA_{t_0}$, e.g. they are biologically older. Similarly, those with the lowest education have smaller differences. In sum, individuals in the most disadvantageous positions have higher BA, a reflection of more rapid aging. An important feature of these results is that the absolute magnitude of estimates from the SEM models are smaller than those from other methods. This is because methods other than SEM generate distributions of $\Delta BA_{t_0}$ with larger variances.

**Table 3.** Linear models for the difference between BA and CA, $\Delta BA_{t_0}$, as a function of sex, race/ethnicity, and education (NHANES 1988–1994).

**Parameter**	**KD**	**MLR**	**PCA**	**SEM: linear**		**SEM: nonlinear**	
				**OF**	**OD**	**OF**	**OD**
CA	0.003	−0.474***	−0.352***	−0.125***	−0.122***	0.183***	0.205***
Female	0.014	−0.501***	−0.409	−0.126	−0.091	−0.366***	−0.408***
Race/ethnicity (ref= NH-white)							
NH Black	3.603***	3.978***	7.049***	3.425***	3.523***	1.916***	2.039***
Mex-Ame	1.114***	1.042***	1.711***	1.050***	1.105***	0.521***	0.579***
Education (ref= less than high school)							
HS	−0.907***	−0.947***	−1.995***	−0.777***	−1.003***	−0.630***	−0.609***
Coll+	−1.927***	−2.380***	−4.493***	−1.793***	−1.920***	−1.277***	−1.283***
*N*	8,759	8,759	8,759	8,759	8,759	8,759	8,759
Adj. *R*^2^	0.109	0.524	0.277	0.246	0.196	0.459	0.496
AIC	53,256.6	57,636.9	64,824.4	50,290.9	52,937.6	43,628.3	44,077.6
BIC	53,313.2	57,693.5	64,881.0	50,347.5	52,994.2	43,684.9	44,134.2

***P-value < 0.000. NH: non-Hispanic; Mex-Ame: Mexican-American; HS: high school; Coll+: some college or more.

## Summary and discussion

### Summary

We propose a generalized method to estimate BA via an SEM. We report four main findings. First, SEM-based estimators relax assumptions about functional forms relating CA and BA, avoid imposing parameter constraints, and facilitate testing of hypotheses about their relations. The empirical results show that a linear functional form with slope different from 1 is more suitable and that parameter constraints imposed by other methods are questionable, weakly justified, and unnecessary.

Second, predicted values of BA from the SEM-based methods are comparable to those obtained from KD (2) but very different from the remaining methods. The OD estimates we computed from NHANES cannot be directly compared with those that utilize epigenetic reads (e.g. PhenoAge) but the proposed method could be easily extended to do so (see below). In an effort to assess how different PhenoAge and OD estimates are, we use the US Health and Retirement Survey (HRS) 2006–2016. This data set includes information to estimate epigenetic clocks and is the basis for computation of PhenoAge estimates (26). Unfortunately, HRS only includes five biomarkers thus placing the OD estimator at a disadvantage relative to those obtained from NHANES. Appendix Section SV shows results. The distributions of BA estimates from PhenoAge and OD are different: Relative to OD, PhenoAge follows a highly smoothed distribution, its location is displaced to the left, and its variance smaller (see Appendix Figures S3 to S5). Clearly, OD and PhenoAge are measuring different objects (Appendix Figure S3). However, even though the OD estimator is based on much less information, it generates BA age patterns similar to those from PhenoAge.

Third, predictions of mortality risk based on models that include the effects of $\Delta BA_{t_0}$, show that SEM methods lead to improved model fit (AIC and BIC metrics), better predictive accuracy (AUC) and, in all cases, imply larger mortality risks than PCA or MLR methods for individuals with the same CA.

Fourth, we show that the OD method allows inclusion of different dimensions of aging and results in models that fit the data more precisely, at least in the case of mortality in NHANES. Furthermore, when we model the difference $\Delta BA_{t_0}$ as a function of gender, educational attainment, and race/ethnicity, we find properly signed and statistically significant estimates of effects, suggesting that physiological deterioration proceeds more rapidly among females, those with lower education, and Mexican-Americans and non-Hispanics Blacks.

### Extensions

The framework proposed here may be extended in several directions. First, one could include additional latent constructs in the SEM to represent different dimensions of BA. For example, one could introduce an epigenetic domain assessed by multiple epigenetic (e.g. tissue dependent) clocks and include them side by side with standard biomarkers representing a partially independent second latent variable representing a different domain. Similarly, a third domain for allostatic load could be added and assessed via inflammatory markers. An SEM model with these two additional latent constructs can further elucidate whether each of them identify complementary, partially overlapping, or distinct underlying deterioration (27). This approach can produce both estimates of multiple BAs (one per domain) and/or a single, unique, estimate that accounts for the independent contributions of separate BAs. Such an estimator integrates information from multiorgan, multicellular, and molecular signatures of gene expression representing cumulative deterioration. An indicator that efficiently combines information from several biological systems should lead to a better understanding of aging, a process driven by the joint influences of multiple interacting domains in the organisms. This approach is in line with systems biology as it integrates data from different domains to gain an understanding of the system as a whole rather than focusing on individual factors (28).

Second, extensions of the OD estimators to outcomes different from mortality may lead to richer characterizations of the latent physiological deterioration that drives senescence. Thus, for example, estimates of BA based on three different and perhaps related outcomes, such as metabolic disorders, cardiovascular events, and disability are likely to reveal more than a single estimate based on only one of them.

Third, an SEM model can also accommodate indicators of distal and proximate factors. Thus, theories of developmental origins of health and disease, DOHaD, (29), and life course epidemiology (30) hypothesize that delayed effects on BA induced by adverse early conditions are important determinants of adult health. Inclusion of exposures across the life course might capture the accumulation of effects posed by chain risk models and will lead to estimates of their impact on rate of senescence and BA (13).

Fourth, the SEM approach can be applied when the data available includes assessments of markers at multiple time points. In this case, the difference between CA and BA becomes a time dependent variable and can provide unique insights on the timing of occurrence of an outcome chosen as anchor. Trajectories of these differences will be more informative and can better elucidate the impact of short-term changes in exposure, interventions, and exogenous shocks.

Fifth, the estimator of biological age we propose, OD, could shed light on competing mechanisms invoked in evolutionary biology to explain senescence and mortality acceleration. Throughout the paper, we conceptualize senescence as has in gerontological research, namely the slope of the force of mortality at older ages, also called the rate of aging or Gompertz slope (13, 14). This parameter reflects the acceleration of physiological deterioration due to cellular death (Hayflick limit), methylation silencing of DNA regions (coding and noncoding) triggered by environmental exposures, misfiring of the HPA axis due to stress, etc. Table 1 shows that the measure of physiological deterioration we use (the structural equation model’s factor scores on which OD depends) accounts for a sizeable fraction of age acceleration at older ages, that is, above and beyond what the Gompertz slope suggests. If the set of biomarkers had been more extensive (and only mildly correlated with those we currently use), then our estimate of BA would account for an even larger fraction of the age acceleration. In the limit, if we could account for all relevant biomarkers, as well as other indicators of systems’ failure, the Gompertz slope in the augmented model should converge to 0. In that case, only the level parameter of the Gompertz function is relevant. This suggests that perhaps it is feasible to assess “components” of the Gompertz slope associated with the physiological systems a researcher is studying.

### Limitations

The paper has two shortcomings. First, it ignores the role played by mediating mechanisms. As we argued before, however, and in contrast to existing methods, the SEM approach can easily accommodate a broad spectrum of mediating mechanisms. Second, we did not test the sensitivity of BA estimates to reductions in the space of biomarkers used by each method. Consequently, we left unexplored the issue of whether different combinations of available biomarkers may influence the robustness of BA estimates. What sort of predictive losses could be associated if a subset of biomarkers is entirely missing? How different would the BA estimates be? Is there a way to adjust for these losses? How portable are these estimates from one study to another?

## Conclusion

The proposed methods to estimate BA have distinctive advantages, including additional flexibility and generalizability, produce estimators with better (mortality-related) predictive power than alternative ones, and admit richer interpretations as indicators of latent deterioration processes and accelerated aging.

## Materials and methods

### Alternative BA estimators

#### Existing estimators

There are three widely used methods to estimate BA from biomarkers as an alternative to CA (2,4, 8). All three methods assume a linear association between CA and biomarkers. The first method is based on multiple linear regression models (heretofore MLR), in which a set of biomarkers are included as covariates of CA and predicted values from the model are interpreted as indicators of BA (4,5). The second approach uses principal components (heretofore PCA) to shrink the space of biomarkers to a small number of principal components while optimizing the amount of variance explained (8). The third method (2) (heretofore KD), is a two-step procedure that first computes bivariate regressions between each biomarker and CA, and then combines parameter estimates from these regressions to estimate BA. All three methods assume a linear association between BA and CA or invoke even stronger constraints (e.g. unit slope) that are inconsistent with theoretical arguments suggesting that aging and senescence are highly nonlinear processes (14) and not always march in lockstep with CA.

#### Two new estimators

We propose two new estimators for BA, which we refer to as the “outcome-free” (OF) and “outcome-dependent” (OD) estimators. Appendix Sections SI to SIII include an extensive and detailed definition of these estimators.

##### OF estimator

This estimator requires two stages. First, we formulate a simple SEM to capture the relations between a latent trait, BA, and observables, namely, CA and relevant biomarkers. The SEM model is general for, in addition to the biomarkers, it allows the inclusion of indicators of other latent individual traits that might be related to exposures influencing BA and its relation to CA and biomarkers. The SEM model assumes that BA is a latent variable measured by observed biomarkers and related to CA by flexible functional forms (Appendix Figure S1). SEM is well suited since it allows representation of the target latent construct (BA), empirical estimates always reflect the variance–covariance structure of the selected biomarkers and CA, and, in addition, the model fully accounts for measurement errors in the explanatory variables, i.e. biomarkers and CA (31). Thus, an SEM model is primed to capture synergies between the biomarkers and CA. Moreover, it does not matter if one uses the original scale of the biomarkers (unstandardized) or standardizes them, the SEM model produces the same factor scores in either case (31). Because the methods we propose (OF and OD) use SEM factor scores to produce BA, it follows that BA is not affected by biomarkers variable scaling.

In a second stage, we compute factors scores and generate estimates of BA that are either linearly or nonlinearly related to CA. SEM’s factor scores [FS(BA)] are unitless quantities that can be used to rank individuals on the unobserved BA and that fully reflect measured variables and their relations (i.e. variance–covariance structure). That is, the FSs can be thought of as a mapping of the vector of unknown BAs onto a vector of real values that preserve the ranking of BA implied by CA and biomarkers. It is up to the researcher to propose models (as we do here) to transform these factor scores into fully scaled units (in our case indicators of biological age). To produce scaled values of BA, we regress the observed CA (linear case) or ln CA (nonlinear case) on the FSs. We then take the predicted values of CA as estimates of BA. In addition, we can test null hypotheses about the values of the linear and nonlinear parameter estimates. When we fail to reject the null that constant and slope are 0 and 1 (linear case) or 1 and 1 (nonlinear case), the SEM model reduces to the very restricted functional forms assumed in the other three methods (see Appendix Sections SI to SIII, for further results).

The SEM at the base of the OF estimators has three advantages. First, it represents explicitly an unobserved, latent, process of physiological deterioration and its parameters. In particular, the parameters relating CA and BA reflect such process and are theoretically meaningful. Second, unlike extant methods, the SEM can include not only biomarkers but also a broad array of observed variables reflecting exposures to risks and medical or individual interventions that could directly affect BA and/or the relation between CA and BA. Third, the SEM model admits not just one, but multiple functional forms and yields information to empirically search a functional form that best fits the data.

##### OD estimator

Computation of OD involves a second stage different from the one supporting the OF variant. Once the SEM model is estimated and the FS’s are computed, we define a health outcome of interest, O, and model it as a function of FS and CA as follows: (i) we first estimate the probability of observing the outcome O as a function of both CA and FS, *pr*(*O*) = Γ(*FZ, CA*) and then as a function solely of *CA*, Γ(*CA*), where Γ(.) is a known, one-to-one function; and (ii) we identify the value of $CA, \widehat{CA}$, that satisfies the identity $\Gamma (FS,\widehat{CA}) = \Gamma (\widehat{CA})$ and we let $\widehat{CA}$*be our estimate of BA*. When *pr*(*O*), one one hand, and CA and FS, on the other, are directly related, $\widehat{CA}\ge CA$. In this case, the difference between the two represents the impact of past accumulated damage which is embedded in the SEM biomarkers.

Although the OD BA estimator defined here uses mortality as outcome, other outcomes might also be suitable. This opens the possibility of testing the existence of biological aging in physiological systems that may be responsible for different health outcomes. Indeed, one could generalize and formulate multiple estimates of BA, each one related to a particular health outcome (see the “Discussion” section). Our OD method is based on estimation of a Gompertz proportional hazard model for the observed mortality experiences in the data (see Appendix Section SII for further details). When we do not control for FS, there is an omitted variable associated with age (underlying deterioration) that influences the estimated Gompertz slope (and age effect): Because FS is a variable positively related to mortality and CA, there is an upward bias in the Gompertz slope. Thus, the difference between the Gompertz slope estimate in a model without controlling for FS and in a model that control for it is a measure of accelerated aging over and above what is reflected by age alone and is attributable to latent deterioration.

### Mortality modeling

To illustrate the use of the new and existing estimators of BA, we model mortality and seek to retrieve effects of CA, BA, and, most importantly, differences between the two. We use a simple parametric (Gompertz) hazard. As most extant data sets, NHANES contains only one set of biomarkers assessments corresponding to the time of the baseline interview. Consequently, we estimate a reduced model with an added fixed covariate, $\Delta BA_{t_0} = BA_{t_0} - CA_{t_0}$, yielding

(1) \begin{eqnarray*} \mu (CA(t))= k \cdot \exp ( \beta \cdot CA_{t_0} ) \cdot \exp (\beta \cdot t) \cdot \exp (\phi \cdot \Delta BA_{t_0} ) \, . \end{eqnarray*}

The parameter *ϕ* is a measure of deterioration as assessed at time *t*_0_, at age $CA_{t_0}$ (see Appendix Section SIII for further details). Although simplified, the model can still reveal the impact of an initial condition, namely, the accumulated latent deterioration at age $CA_{t_0}$, which is added to the effect of CA. As shown in Table 2, the fit of [1] to the NHANES data is best in AIC and BIC metrics when using a linear SEM (OF) to predict $BA_{t_0}$. Estimates of the area under the receiver operating characteristics curve (ROC curve) show that the linear and nonlinear SEM models perform better than other methods (Appendix Table S8). (All analyses were conducted using the R statistical software version 4.0.1 R Core Team. 2020. Available: http://www.R-project.org/.)

### Data

We use data from the third National Health and Nutrition Examination Survey (NHANES III), a nationally representative, cross-sectional study conducted by the National Center for Health Statistics between 1988 and 1994. NHANES III data were collected from at-home interviews and examinations taking place at a Mobile Examination Center. In this paper, we selected people aged 30 to 75 y to focus on the adult population and excluded older adults (aged 75+) to reduce mortality selection bias. Of the 12,517 NHANES subjects, aged 30 to 75 y, our final analytic sample included 9,389 participants with complete information on the biomarkers of interest.

### Biomarkers

We selected nine biomarkers representing underlying physiological functioning of seven major systems (Appendix Table S1). These markers have been shown to be associated with adult mortality and survival (3) and have been used routinely as clinical markers of underlying diseases (32). Details of data collection and assay processing for blood biomarkers are elsewhere (see NCHS reference manuals at https://wwwn.cdc.gov/nchs/nhanes/nhanes3/manualsandreports.aspx). As noted in Appendix Section SB.1, it does not matter if one uses standardized or unstandardized biomarkers, the SEM model produces the same factor scores in either case.

### Mortality

The NHANES III (1988–1994) includes a mortality follow-up consisting of individual observations linked to National Death Index records through 2015. We excluded deaths due to HIV, violence, or accidents as these are unlikely to be due to an age-related process. Data on mortality was available for all NHANES participants covering an interval of time between 21 and 27 y as the baseline information was collected over a 6-y period, between 1988 and 1994 with a mid-point at about 2003. Our estimates of life expectancy at age 65, E(65), are similar to those from national-level data from the National Center for Health Statistics (NCHS) in 2003 (33). For example, we estimated females’ E(65) to be 19.9 y versus 19.7 y in NCHS and 16.8 for males, identical to NCHS’s estimates.

## Supplementary Material

pgac135_Supplemental_File

## Data Availability

Data available here. https://gitlab.com/hirambeltran/physiological-clocks/-/blob/7220e56693f5a67e5f036646441fff76e69a9e4d/NHANES3_results.dta.

## References

[1] Jackson SH , Weale MR, Weale RA. 2003. Biological age—what is it and can it be measured?. Arch Gerontol Geriatr. 36(2):103–115. 12849085 10.1016/s0167-4943(02)00060-2

[2] Klemera P , Doubal S., 2006. A new approach to the concept and computation of biological age. Mech Ageing Dev. 127(3): 240–248. 16318865 10.1016/j.mad.2005.10.004

[3] Levine ME . 2013. Modeling the rate of senescence: can estimated biological age predict mortality more accurately than chronological age?. J Gerontol A Biol Sci Med Sci. 68(6):667–674. 23213031 10.1093/gerona/gls233 PMC3660119

[4] Voitenko VP , Tokar AV., 1983. The assessment of biological age and sex differences of human aging. Exp Aging Res. 9(4): 239–244. 6667707 10.1080/03610738308258458

[5] Weale RA. , 1997. Human biological decline and mortality rates. Mech Ageing Dev. 97(1): 55–72. 9223126 10.1016/s0047-6374(97)01906-4

[6] Levine ME , Crimmins EM., 2018. Is 60 the new 50? Examining changes in biological age over the past two decades. Demography. 55(2): 387–402. 29511995 10.1007/s13524-017-0644-5 PMC5897168

[7] Brown PJ , et al., 2018. Biological age, not chronological age, is associated with late-life depression. J Gerontol A Biol Sci Med Sci. 73(10): 1370–1376. 28958059 10.1093/gerona/glx162 PMC6132120

[8] Nakamura E , Miyao K, Ozeki T., 1988. Assessment of biological age by principal component analysis. Mech Ageing Dev. 46(1–3): 1–18. 3226152 10.1016/0047-6374(88)90109-1

[9] Waziry R , et al., 2019. Quantification of biological age as a determinant of age-related diseases in the Rotterdam study: a structural equation modeling approach. Eur J Epidemiol. 34(8): 793–799. 30993509 10.1007/s10654-019-00497-3

[10] Horvath S. , 2013. DNA methylation age of human tissues and cell types. Genome Biol. 14(10): 1–20. 10.1186/gb-2013-14-10-r115 PMC4015143 24138928

[11] Belsky DW et al. , 2015. Quantification of biological aging in young adults. Proc Natl Acad Sci U S A. 112(30): E4104–E4110. 26150497 10.1073/pnas.1506264112 PMC4522793

[12] Jansen R et al. , 2021. An integrative study of five biological clocks in somatic and mental health. Elife. 10: e59479. 33558008 10.7554/eLife.59479 PMC7872513

[13] Finch C. 2007. The biology of human longevity : inflammation, nutrition, and aging in the evolution of life spans. 1st edn, Burlington, MA: Academic Press, p. 626.

[14] Finch C , Kirkwood TBL. 2000. Chance, development, and aging. New York (NY): Oxford University Press, p. 278.

[15] Beltrán-Sánchez H , Harhay MO, Harhay MM, McElligott S., 2013. Prevalence and trends of the metabolic syndrome in the adult US population, 1999–2010. J Am Coll Cardiol. 62(8): 697–703. 23810877 10.1016/j.jacc.2013.05.064 PMC3756561

[16] Charlesworth B . 1994. Evolutionary mechanisms of senescence. In: Rose MR, Finch CE, editors. Genetics and Evolution of Aging. Contemporary Issues in Genetics and Evolution. Dordrecht: Springer-Science+Business Media, B.V. p. 13–21.

[17] Finch C. 1990. Longevity, senescence, and the genome. The John D and Catherine T MacArthur Foundation series on mental health and development, Chicago (IL): University of Chicago Press, p. 922.

[18] Hamilton WD. , 1966. The moulding of senescence by natural selection. J Theor Biol. 12(1): 12–45. 6015424 10.1016/0022-5193(66)90184-6

[19] Medawar PB. , 1946. Old age and natural death. Mod Quart. 1: 30–56.

[20] Williams GC. , 1957. Pleiotropy, natural selection and the evolution of senescence. Evolution. 11: 398–411.

[21] Bongaarts J. , 2005. Long-range trends in adult mortality: models and projection methods. Demography. 42(1): 23–49. 15782894 10.1353/dem.2005.0003

[22] Bongaarts J. , 2009. Trends in senescent life expectancy. Popul Stud. 63(3): 203–213. 10.1080/00324720903165456 PMC2772142 19851933

[23] Colchero F et al. 2021. The long lives of primates and the “invariant rate of ageing” hypothesis. Nat Commun. 12(1):3666. 34135334 10.1038/s41467-021-23894-3 PMC8209124

[24] Corti MC , Guralnik JM, Salive ME, Sorkin JD., 1994. Serum-albumin level and physical-disability as predictors of mortality in older persons. JAMA. 272(13): 1036–1042. 8089886

[25] Finch CE , Pike MC, Witten M., 1990. Slow mortality rate accelerations during aging in some animals approximate that of humans. Science. 249(4971): 902–905. 2392680 10.1126/science.2392680

[26] Levine ME et al. , 2018. An epigenetic biomarker of aging for lifespan and healthspan. Aging (Albany NY). 10(4): 573. 29676998 10.18632/aging.101414 PMC5940111

[27] McCrory C et al. , 2020. Epigenetic clocks and allostatic load reveal potential sex-specific drivers of biological aging. J Gerontol A Biol Sci Med Sci. 75(3): 495–503. 31603985 10.1093/gerona/glz241

[28] Zierer J , Menni C, Kastenmuller G, Spector TD., 2015. Integration of ‘omics’ data in aging research: from biomarkers to systems biology. Aging Cell. 14(6): 933–44. 26331998 10.1111/acel.12386 PMC4693464

[29] Gluckman PD , Hanson MA. The developmental origins of health and disease. Cambridge (MA): Cambridge University Press.

[30] Ben-Shlomo Y , Kuh D., 2002. A life course approach to chronic disease epidemiology: conceptual models, empirical challenges and interdisciplinary perspectives. Int J Epidemiol. 31(2): 285–293. 11980781

[31] Bollen KA . 1989. Structural equations with latent variables. Wiley series in probability and mathematical statistics applied probability and statistics. New York (NY): Wiley, p. 514.

[32] Alberti KG et al. , 2009. Harmonizing the metabolic syndrome: a joint interim statement of the International Diabetes Federation Task Force on Epidemiology and Prevention; National Heart, Lung, and Blood Institute; American Heart Association; World Heart Federation; International Atherosclerosis Society; and International Association for the Study of Obesity. Circulation. 120(16): 1640–1645. 19805654 10.1161/CIRCULATIONAHA.109.192644

[33] Arias E. , 2006. United States life tables, 2003. Natl Vital Stat Rep. 54(14): 1–40. 16681183

