# Supplementary material for: Modeling biological age and its link with the aging process
Source: PNAS Nexus. 2022 Jul 26;1(3):pgac135. doi: 10.1093/pnasnexus/pgac135 (PMC9896935; doi:10.1093/pnasnexus/pgac135)
Supplement: pgac135_Supplemental_File [file pgac135_supplemental_file.pdf]

Modeling biological age and its link with the aging process

Hiram Beltrán-Sánchez[1]
Alberto Palloni[2,3]
Yiyue Huangfu[2]
Mary C. McEniry[2]

Abstract

Differences in health status at older ages are a result of genetic predispositions and physiological responses to exposure accumulation over the lifespan. These vary across individuals and lead to health status heterogeneity as people age. Chronological age (CA) is a standard indicator that reflects overall risks of morbidity and mortality. However, CA is only a crude proxy for individuals' latent physiological deterioration. An alternative to CA is biological age, (BA), an indicator of accumulated age-related biological change reflected in markers of major physiological systems. We propose and validate two BA estimators that improve upon existing ones. These estimators: (i) are based on a structural equation model (SEM) that represent the relation between BA and CA, (ii) circumvent the need to impose arbitrary assumptions about the relation between CA and BA, and (iii) provide tools to empirically test the validity of assumptions the researcher may wish to invoke. We use the US National Health and Nutrition Examination Survey 1988-1994 and compare results with three commonly used methods to compute BA (principal components--PCA, multiple regression--MLR, and Klemera-Doubal's method--KD). We show that SEM-based estimates of BA differ significantly from those generated by PCA and MLR and are comparable to, but have better predictive power than KD's. The proposed estimators are flexible, allow testing of assumptions about functional forms relating BA and CA, and admit a rich interpretation as indicators of accelerated aging.

Significance statement

Several methods have been proposed to estimate BA from available biomarkers, all of them rely on linear regression approaches, and all constrain the functional form of the relation between CA and BA. SEM-based estimators relax assumptions about functional forms and do not impose parameter constraints. Our empirical results suggest that parameter constraints imposed by other methods are unnecessary and could produce misleading inferences. One of the proposed BA estimators, outcome-dependent, is the only one that suggests faster underlying physiological deterioration relative to CA. This is consistent with accelerated biological aging and senescent mortality. SEM-based estimators could provide empirical evidence to discriminate between competing mechanisms invoked in evolutionary biology to explain senescence and mortality acceleration

[1] Fielding School of Public Health and California Center for Population Research, UCLA
[2] Center for Demography of Health and Aging, University of Wisconsin–Madison
[3] Consejo Superior de Investigaciones Cientificas (CSIC, Madrid, Spain)

Appendix

Section I. Transforming SEM factor scores into estimates of BA: outcome-free estimator

Factors scores from an SEM model, FS(BA), are unitless measures of the latent variable BA. To work with a suitably scaled BA in the lineal model we regress the observed values of CA on FS(BA)

$$CA = \alpha_0 + \alpha_1 \cdot \text{FS(BA)} + \varepsilon$$

and then compute estimates of BA, $\widehat{BA} = \alpha_0 + \alpha_1 FS(BA)$.

A similar procedure is implemented in the non-linear cases except that we estimate the regression

$$\ln CA = \alpha_0' + \alpha_1' \cdot \text{FS(BA)} + \varepsilon$$

and predict values $= \exp(\alpha_0' + \alpha_1' \cdot FS(BA))$. Values of estimates of $\alpha_0,\ \alpha_1,\ {\alpha_0}',\ \text{and}\ \ {\alpha_1}',$ are shown in the table below.

Parameters of the linear association between factors scores and CA for the outcome-free estimator (OF)

	SEM: Linear		SEM: Non-Linear[1]	
	Estimate	95% CI	Estimate	95% CI
Males				
Intercept	6.69	[7.22,6.16]	0.55	[0.55,0.59]
FS(BA)	49.26	[49.83,48.68]	0.98	[0.98,0.99]
Females				
Intercept	6.24	[6.72,5.76]	0.51	[0.51,0.55]
FS(BA)	37.05	[37.45,36.66]	0.75	[0.75,0.76]

FS(BA) factors scores from an SEM model

[1] The linear equation for the SEM non-linear is given by: $\ln CA = \alpha_0' + \alpha_1' \cdot \text{FS(BA)} + \varepsilon$

Section II. Transforming SEM factor scores into estimates of BA: outcome-dependent estimator

We estimate two Gompertz hazard models, one depending on CA and the other depending on CA and FS(BA):

$\mu^1(CA) = \mu_o^1 \cdot \exp(\beta^1 \text{t}) \cdot \exp(\beta^1 \text{CA})$ (1)

and

$\mu^2(CA) = \mu_o^2 \cdot \exp(\beta^2 \text{t}) \cdot \exp(\beta^2 \text{CA}) \cdot \exp(\gamma \cdot \text{FS(BA)})$ (2)

where *t* is the time elapsed since an individual was first observed. For each observation *i* the estimate of BA, $\widehat{BA}$, will be defined as the value of CA, CA" that satisfies the following equality:

We take the predicted value of $\mu^2(CA\ )$ and use it in (1) instead of $\mu^1(CA)$ to solve for BA*:

$\ln \mu^2(CA) = \ln \mu_o^1 + \beta^1 t + \beta^1 \text{BA*}$

using the expression (2) for $\mu^2(CA)$ in (1) and then solving for BA* we get

$\ln\mu_o^2 + \beta^2 t + \beta^2 CA + \gamma \cdot \mathrm{FS(BA)} = \ln\mu_o^1 + \beta^1 t + \beta^1$BA*

BA* = $(\ln\mu_o^2 + \beta^2 t + \beta^2 \mathrm{CA} + \gamma \cdot \mathrm{FS(BA)} - \ln\mu_o^1 - \beta^1 t)/\beta^1$

BA*=$(\ln(\mu_o^2/\mu_o^1) + \beta^2 CA + (\beta^2 - \beta^1) \cdot t + \gamma \cdot \mathrm{FS(BA)})/\beta^1$

If after accounting for CA, there is no relation between FS(BA) and mortality, $\widehat{BA} \sim CA$. Unlike the outcome-free estimator, this derivation does not depend on a simple transformation (lineal or loglinear) that coverts CA 's into BA. However, it is bound to either a linear or log linear functional form at the level of SEM.

Section III. Mortality modelling

To illustrate the use of the new and existing estimators of BA, we model mortality and seek to retrieve effects of CA, BA and, most importantly, differences between the two. We use a simple parametric (Gompertz) hazard[1]

$$\mu(CA(t)) = K \cdot \exp(\beta \cdot CA(t)) \quad (1)$$

where t is the number of years since the first observation and $K = \exp\left(\sum \alpha_j Z_j\right)$ with Z_j being the jth individual covariate. Note that CA(t) is a time varying covariate that depends on the time elapsed since the first interview, t. Thus,

$$CA(t) = CA_{t_o} + t \quad (2)$$

And, upon replacing this expression into CA(t) we get

$$\mu(CA(t)) = K \cdot \exp\left(\beta \cdot CA_{t_o}\right) \cdot \exp(\beta \cdot t) \quad (3)$$

This hazard model must be estimated constraining the coefficient of CA_{t_o} to be identical to the coefficient of t. This coefficient, β, approximates the standard Gompertz slope and has been traditionally interpreted as the rate of aging. If CA is measured in years and assessments of BA were available yearly, we could define

ΔBA(t) = $\mathrm{BA(t)} - CA(t)$ and the hazard model becomes

$$\mu(CA(t)) = K \cdot \exp\left(\beta \cdot CA_{t_o}\right) \cdot \exp(\beta \cdot t) \cdot \exp(\gamma \cdot \Delta\mathrm{BA(t)}) \quad (4)$$

where the quantity $\Delta\mathrm{BA(t)}$ is as an indicator of accumulated "excess" individual's physiological deterioration relative to that associated with the passage of time (t) and the parameter γ is a measure of its impact on mortality.[2] As most extant data sets, NHANES contains only one set of biomarkers

[1] In what follows BA stands for any of the three alternative estimates of BA (two outcome-free and one outcome-dependent estimate).

[2] This continuous version of the model simplifies notation. It is a highly unrealistic representation since there are no data sets in which biomarkers are assessed more than once or twice, let alone yearly. Thus, the value of ΔBA(t)) may be available only once (as in our case) and at most in one or two additional points in time. A discrete version of (5) can be derived from first principles and could be used when the researcher has access to biomarker assessments at multiple time nodes separate by unequal time periods.

assessments corresponding to the time of the baseline interview. Consequently, model (4) must be reduced to model (3) with an added fixed covariate, $\Delta\mathrm{BA}t_o = \mathrm{BA}(t_o) - CA(t_o)$, yielding

$$\mu(CA(t)) = K \cdot \exp\left(\beta \cdot CA_{t_o}\right) \cdot \exp(\varphi \cdot \Delta\mathrm{BA}t_o) \cdot \exp(\beta \cdot t) \tag{5}$$

The parameter φ is now a measure of the mortality effect of accumulated deterioration at *time* t_o and age CA_{t_o} .

Section IV. Application: Accelerated aging by gender, race/ethnicity and education

The goal of this exercise is to assess accelerated aging by education and race/ethnicity since these are two of the main individual characteristics associated with poor health. For example, there is a large body of literature documenting the impact of social determinants of health whereby individuals with low socioeconomic status (e.g., low education) and minority populations (e.g., Non-Hispanic Blacks) tend to have worse health risk factors and health outcomes, including lower life expectancy relative to Non-Hispanic whites (Institute of Medicine and National Research Council 2013). Our results in Table 3 are consistent with the literature: Non-Hispanic Blacks and Mexican American have positive values of the difference (BA-CA) and their CA's are smaller than their BA's (i.e., biologically older). Similarly, those with the lowest levels of education experience the lowest levels of the difference. In sum, individuals in the most disadvantageous positions have higher BA, a reflection of more rapid aging.

Information on race/ethnicity is self-reported by survey respondents and it does not represent ancestry (or heritage). We focus on the three main groups identified in the data set available to us (NHANES). For example, the race/ethnicity distribution of the sample is 42.6% Non-Hispanic white, 26.9% Non-Hispanic Black, 26.2% Mexican-American and only 4.3% of other race/ethnicities. The NHANES sample size is not large enough to study other race/ethnicity groups. Moreover, the data set contains no information that would allow us to assess whether non-Hispanic whites (or any other race/ethnicity groups) are all of the same ethnicity.

Section V. Contrast between PhenoAge and OD

The following is a comparison of the steps followed in the construction of PhenoAge and OD.

PhenoAge	SEM linear OD
Selection of biomarkers: a subset of biomarkers is selected using a Cox (penalized) model and an arbitrary defined lambda criterion.	We don't preselect biomarkers. The biomarkers we include in the SEM model must be theoretically justified as they reflect possibly multiple latent constructs.
Phenotypic age (first stage PhenoAge): Using the preselected biomarkers a Gompertz models is estimated with data on death. The predicted Gompertz survival inverse function is used to compute an estimated age at death.	Predict SEM factor scores (FS). These FS are scaleless scores ranking individuals on the latent, unobserved variable. These values are independent of mortality.

PhenoAge: a penalized regression is used to estimate the relation between (*predicted*) Phentoypic age as a function of CpGs. The *predicted* values from this regression are the final PhenoAge values. PhenoAge relies on predicted values to generate subsequent predicted values, thus creating a sort of regression-to-mean effect.	Estimate two simple death models for mortality. The first model only includes chronological age (CA) as a predictor. The second includes CA and predicted factor scores FS.<br><br>Convert the predicted hazard rate for age x in the second model into an age, x', in the first model that yields the same hazard rate identified at age x in the second model. This is our final estimate OD. When the relation between mortality and factor scores is positive, the difference (x' – x) > 0 represents the increase in "aging" induced by deterioration reflected in the biomarkers.

Appendix Table S1. Descriptive values of the biomarkers for the total population and for women and men

Biomarker	Total Population (N=9,197)				Women (N=4,842)				Men (N=4,355)			
	mean	sd	min	max	mean	sd	min	max	mean	sd	min	max
Age	49.7	13.6	30.0	75.0	49.2	13.6	30.0	75.0	50.3	13.6	30.0	75.0
Metabolic function												
1. Glycated hemoglobin (%)	5.6	1.2	3.3	16.1	5.6	1.3	3.3	16.1	5.7	1.2	3.3	15.7
2. Serum total cholesterol (mg/dL)	210.8	43.4	79.0	501.0	211.3	44.8	94.0	464.0	210.1	41.7	79.0	501.0
Cardiac function												
3. Systolic blood pressure	124.9	19.9	58.0	254.7	122.7	20.9	58.0	230.7	127.3	18.3	80.0	254.7
Lung function												
4. Forced expiratory volume (mL)	2523.2	821.5	173.3	5031.8	2331.2	776.4	173.3	4267.8	2736.8	817.6	292.0	5031.8
Kidney function												
5. Serum creatinine (mg/dL)	1.1	0.4	0.3	13.9	1.0	0.3	0.3	10.1	1.2	0.4	0.4	13.9
6. Serum urea nitrogen (mg/dL)	14.3	5.3	2.0	86.0	13.4	5.1	2.0	79.0	15.3	5.3	2.0	86.0
Liver function												
7. Serum alkaline phosphatase SI (U/L)	87.1	33.1	17.0	952.0	86.4	33.6	18.0	721.0	88.0	32.4	17.0	952.0
8. Serum albumin (g/dL)	4.1	0.4	2.0	5.7	4.0	0.3	2.0	5.5	4.2	0.4	2.2	5.7
Immune Function and inflammation												
9. C-reactive protein (mg/dL)	0.5	0.8	0.2	25.2	0.6	0.9	0.2	25.2	0.4	0.7	0.2	19.8

Note: sd=standard deviation, min=minimum and max=maximum.

Appendix Table S2. Full set of estimates from SEM models for the total population (N=9,197).

	Coefficient	95%CI			Coefficient	95%CI	
		lower	upper			lower	upper
**Linear**				**NON-Linear**			
**Slopes of biomarkers in relation to BA**							
BA=~GlyHemog	1.00	1.00	1.00	BA=~GlyHemog	1.00	1.00	1.00
BA=~Cholesterol	0.91	0.85	0.97	BA=~Cholesterol	0.92	0.86	0.98
BA=~Sys blood press	1.57	1.49	1.66	BA=~Sys blood press	1.55	1.47	1.63
BA=~Forced exp vol	-2.15	-2.25	-2.05	BA=~Forced exp vol	-2.12	-2.22	-2.02
BA=~Creatinine	0.68	0.63	0.74	BA=~Creatinine	0.66	0.61	0.72
BA=~Urea	1.07	1.00	1.13	BA=~Urea	1.04	0.97	1.10
BA=~Phosphatase	0.78	0.72	0.83	BA=~Phosphatase	0.78	0.72	0.83
BA=~Albumin	-0.66	-0.71	-0.60	BA=~Albumin	-0.65	-0.71	-0.60
BA=~CRP	0.49	0.44	0.54	BA=~CRP	0.49	0.43	0.54
**Latent component**							
BA~age	0.02	0.02	0.02	BA~ln.age	1.03	0.98	1.08
**Variance components**							
GlyHemog	0.89	0.87	0.90	GlyHemog	0.88	0.86	0.90
Cholesterol	0.91	0.89	0.92	Cholesterol	0.90	0.88	0.92
Sys blood press	0.72	0.70	0.73	Sys blood press	0.72	0.70	0.74
Forced exp vol	0.47	0.46	0.49	Forced exp vol	0.48	0.46	0.49
Creatinine	0.95	0.93	0.97	Creatinine	0.95	0.93	0.97
Urea	0.87	0.85	0.89	Urea	0.87	0.86	0.89
Phosphatase	0.93	0.91	0.95	Phosphatase	0.93	0.91	0.95
Albumin	0.95	0.93	0.97	Albumin	0.95	0.93	0.97
CRP	0.97	0.95	0.99	CRP	0.97	0.95	0.99
BA	0.03	0.03	0.04	BA	0.04	0.03	0.04
Age	184.65	184.65	184.65	ln.age	0.08	0.08	0.08

Note: GlyHemog=Glycated hemoglobin (%); Cholesterol=Serum total cholesterol (mg/dL); Sys blood press =Systolic blood pressure; Forced exp vol=Forced expiratory volume (mL); Creatinine =Serum creatinine (mg/dL); Urea=Serum urea nitrogen (mg/dL); Phosphatase=Serum alkaline phosphatase SI (U/L); Albumin=Serum albumin (g/dL); CRP= C-reactive protein (mg/dL). Non-linear model: BA = age^r which implies ln(BA) = r*ln(age)

Appendix Table S3. Full set of estimates from SEM models for females (N=4,355).

	Coefficient	95%CI			Coefficient	95%CI	
		lower	upper			lower	upper
**Linear**				**NON-Linear**			
**Slopes of biomarkers in relation to BA**							
BA=~GlyHemog	1.00	1.00	1.00	BA=~GlyHemog	1.00	1.00	1.00
BA=~Cholesterol	1.18	1.10	1.27	BA=~Cholesterol	1.18	1.10	1.26
BA=~Sys blood press	1.58	1.49	1.68	BA=~Sys blood press	1.57	1.47	1.66
BA=~Forced exp vol	-1.93	-2.05	-1.82	BA=~Forced exp vol	-1.91	-2.02	-1.80
BA=~Creatinine	0.64	0.57	0.71	BA=~Creatinine	0.62	0.56	0.69
BA=~Urea	1.17	1.09	1.25	BA=~Urea	1.14	1.06	1.22
BA=~Phosphatase	0.90	0.83	0.98	BA=~Phosphatase	0.90	0.83	0.98
BA=~Albumin	-0.32	-0.38	-0.26	BA=~Albumin	-0.31	-0.37	-0.25
BA=~CRP	0.37	0.31	0.43	BA=~CRP	0.37	0.31	0.43
**Latent component**							
BA~age	0.02	0.02	0.02	BA~ln.age	1.16	1.10	1.23
**Variance components**							
GlyHemog	0.86	0.84	0.89	GlyHemog	0.86	0.83	0.88
Cholesterol	0.80	0.78	0.83	Cholesterol	0.80	0.78	0.83
Sys blood press	0.65	0.63	0.67	Sys blood press	0.65	0.63	0.67
Forced exp vol	0.48	0.46	0.50	Forced exp vol	0.48	0.46	0.50
Creatinine	0.94	0.92	0.97	Creatinine	0.94	0.92	0.97
Urea	0.81	0.79	0.83	Urea	0.81	0.79	0.84
Phosphatase	0.89	0.86	0.91	Phosphatase	0.88	0.86	0.91
Albumin	0.99	0.96	1.01	Albumin	0.99	0.96	1.01
CRP	0.98	0.95	1.01	CRP	0.98	0.95	1.01
BA	0.04	0.03	0.04	BA	0.04	0.03	0.04
Age	184.65	184.65	184.65	ln.age	0.08	0.08	0.08

Note: GlyHemog=Glycated hemoglobin (%); Cholesterol=Serum total cholesterol (mg/dL); Sys blood press =Systolic blood pressure; Forced exp vol=Forced expiratory volume (mL); Creatinine =Serum creatinine (mg/dL); Urea=Serum urea nitrogen (mg/dL); Phosphatase=Serum alkaline phosphatase SI (U/L); Albumin=Serum albumin (g/dL); CRP= C-reactive protein (mg/dL). Non-linear model: BA = age^r which implies ln(BA) = r*ln(age)

Appendix Table S4. Full set of estimates from SEM models for males (N=4,842).

	Coefficient	95%CI			Coefficient	95%CI	
		lower	upper			lower	upper
**Linear**				**NON-Linear**			
**Slopes of biomarkers in relation to BA**							
BA=~GlyHemog	1.00	1.00	1.00	BA=~GlyHemog	1.00	1.00	1.00
BA=~Cholesterol	0.51	0.42	0.60	BA=~Cholesterol	0.53	0.44	0.62
BA=~Sys blood press	1.57	1.43	1.71	BA=~Sys blood press	1.53	1.40	1.67
BA=~Forced exp vol	-2.53	-2.73	-2.32	BA=~Forced exp vol	-2.47	-2.68	-2.27
BA=~Creatinine	0.74	0.65	0.84	BA=~Creatinine	0.73	0.63	0.82
BA=~Urea	0.94	0.84	1.05	BA=~Urea	0.91	0.80	1.01
BA=~Phosphatase	0.59	0.50	0.69	BA=~Phosphatase	0.59	0.50	0.68
BA=~Albumin	-1.20	-1.32	-1.08	BA=~Albumin	-1.19	-1.31	-1.07
BA=~CRP	0.67	0.57	0.77	BA=~CRP	0.66	0.57	0.75
**Latent component**							
BA~age	0.02	0.02	0.02	BA~ln.age	0.88	0.81	0.94
**Variance components**							
GlyHemog	0.92	0.89	0.95	GlyHemog	0.91	0.89	0.94
Cholesterol	0.98	0.95	1.01	Cholesterol	0.98	0.95	1.01
Sys blood press	0.80	0.77	0.82	Sys blood press	0.80	0.77	0.82
Forced exp vol	0.47	0.45	0.50	Forced exp vol	0.48	0.45	0.50
Creatinine	0.95	0.93	0.98	Creatinine	0.95	0.93	0.98
Urea	0.93	0.90	0.95	Urea	0.93	0.90	0.96
Phosphatase	0.97	0.94	1.00	Phosphatase	0.97	0.94	1.00
Albumin	0.88	0.85	0.91	Albumin	0.88	0.85	0.91
CRP	0.96	0.93	0.99	CRP	0.96	0.93	0.99
BA	0.03	0.02	0.03	BA	0.03	0.02	0.03
Age	184.65	184.65	184.65	ln.age	0.08	0.08	0.08

Note: GlyHemog=Glycated hemoglobin (%); Cholesterol=Serum total cholesterol (mg/dL); Sys blood press =Systolic blood pressure; Forced exp vol=Forced expiratory volume (mL); Creatinine =Serum creatinine (mg/dL); Urea=Serum urea nitrogen (mg/dL); Phosphatase=Serum alkaline phosphatase SI (U/L); Albumin=Serum albumin (g/dL); CRP= C-reactive protein (mg/dL). Non-linear model: BA = age^r which implies ln(BA) = r*ln(age)

Appendix Table S5. Partial set of estimates and measures of fit SEM by sex.

	Total Population (N=9,197)		Females (N=4,842)		Males (N= 4,355)	
	Linear	Non-Linear	Linear	Non-Linear	Linear	Non-Linear
**Latent component**						
BA~age	0.021	---	0.024	---	0.018	---
BA~ln.age	---	1.030	---	1.162	---	0.875
**Slopes of biomarkers in relation to BA**						
GlyHemog	1.000	1.000	1.000	1.000	1.000	1.000
Cholesterol	0.913	0.868	1.183	1.087	0.514	0.528
Sys blood press	1.574	1.453	1.584	1.475	1.571	1.421
Forced exp vol	-2.152	-1.963	-1.934	-1.770	-2.525	-2.303
Creatinine	0.681	0.642	0.640	0.602	0.745	0.702
Urea	1.069	0.932	1.168	1.032	0.945	0.792
Phosphatase	0.777	0.784	0.902	0.905	0.594	0.608
Albumin	-0.659	-0.642	-0.316	-0.332	-1.201	-1.155
CRP	0.489	0.504	0.367	0.407	0.670	0.658
LogLik	-112,017.9	-112,062.0	-58,266.3	-58,287.9	-53,480.0	-53,503.3
AIC	224,073.8	224,161.9	116,570.6	116,613.7	106,998.0	107,044.5
BIC	224,209.2	224,297.3	116,693.8	116,736.9	107,119.2	107,165.7

Notes:

i. All coefficients reached a p-value <0.001 and for simplicity we did not include significance levels (see Appendix Tables 2-4 for the full tables).

ii. GlyHemog=Glycated hemoglobin (%); Cholesterol=Serum total cholesterol (mg/dL); Sys blood press =Systolic blood pressure; Forced exp vol=Forced expiratory volume (mL); Creatinine =Serum creatinine (mg/dL); Urea=Serum urea nitrogen (mg/dL); Phosphatase=Serum alkaline phosphatase SI (U/L); Albumin=Serum albumin (g/dL); CRP= C-reactive protein (mg/dL). LogLik=log-likelihood.

Non-linear model: BA = k*CA^r which implies ln(BA) =ln(k)+ r*ln(age).

Appendix Table S6. Regression results for the association between BA and CA (panel A), and the difference between BA and CA vs CA (Panel B)

Panel A: BA vs CA

		KD	MLR	PCA	SEM, Lin (outc-free)	SEM, Non-Lin (outc-free)	SEM, Lin (outc-dep)	SEM, Non-Lin (outc-dep)
Male	(Intercept)	-0.0060	26.1298	18.9907	6.6888	6.2157	-12.1389	-13.1464
		CI [-0.6321, 0.6201]	CI [25.3568, 26.9028]	CI [17.7795, 20.2019]	CI [6.1630, 7.2146]	CI [5.6070, 6.8244]	CI [-12.5025, -11.7753]	CI [-13.5177, -12.7750]
	age	1.0001	0.4801	0.6222	0.8669	0.8717	1.1868	1.2048
		CI [0.9881, 1.0121]	CI [0.4653, 0.4950]	CI [0.5989, 0.6454]	CI [0.8568, 0.8770]	CI [0.8601, 0.8834]	CI [1.1798, 1.1938]	CI [1.1977, 1.2120]
	Adjusted R2	0.8594	0.4803	0.3873	0.8669	0.8309	0.9623	0.9619
	AIC	27202.9334	29037.4996	32946.3632	25682.9913	26957.6625	22472.3429	22655.9903
	BIC	27222.0686	29056.6347	32965.4983	25702.1265	26976.7976	22491.4780	22675.1255

		KD	MLR	PCA	SEM, Lin (outc-free)	SEM, Non-Lin (outc-free)	SEM, Lin (outc-dep)	SEM, Non-Lin (outc-dep)
Female	(Intercept)	0.0730	21.5774	16.7431	6.2401	5.8255	-12.0379	-13.3572
		CI [-0.4763, 0.6223]	CI [20.8627, 22.2920]	CI [15.6609, 17.8254]	CI [5.7611, 6.7191]	CI [5.2811, 6.3699]	CI [-12.3512, -11.7246]	CI [-13.6813, -13.0331]
	age	0.9986	0.5617	0.6599	0.8733	0.8772	1.1772	1.2006
		CI [0.9878, 1.0093]	CI [0.5477, 0.5757]	CI [0.6387, 0.6810]	CI [0.8639, 0.8826]	CI [0.8666, 0.8879]	CI [1.1710, 1.1833]	CI [1.1943, 1.2070]
	Adjusted R2	0.8726	0.5614	0.4351	0.8732	0.8433	0.9669	0.9660
	AIC	29626.8085	32172.4619	36187.6110	28301.7533	29539.6813	24195.4993	24523.2119
	BIC	29646.2606	32191.9140	36207.0632	28321.2055	29559.1334	24214.9515	24542.6640

Panel B: difference between BA and CA vs CA

		KD	MLR	PCA	SEM, Lin (outc-free)	SEM, Non-Lin (outc-free)	SEM, Lin (outc-dep)	SEM, Non-Lin (outc-dep)
Male	(Intercept)	-0.0060	26.1298	18.9907	6.6888	6.2157	-12.1389	-13.1464
		CI [-0.6321, 0.6201]	CI [25.3568, 26.9028]	CI [17.7795, 20.2019]	CI [6.1630, 7.2146]	CI [5.6070, 6.8244]	CI [-12.5025, -11.7753]	CI [-13.5177, -12.7750]
	age	0.0001	-0.5199	-0.3778	-0.1331	-0.1283	0.1868	0.2048
		CI [-0.0119, 0.0121]	CI [-0.5347, -0.5050]	CI [-0.4011, -0.3546]	CI [-0.1432, -0.1230]	CI [-0.1399, -0.1166]	CI [0.1798, 0.1938]	CI [0.1977, 0.2120]
	Adjusted R2	-0.0002	0.5200	0.1889	0.1329	0.0960	0.3873	0.4216
	AIC	27202.9334	29037.4996	32946.3632	25682.9913	26957.6625	22472.3429	22655.9903
	BIC	27222.0686	29056.6347	32965.4983	25702.1265	26976.7976	22491.4780	22675.1255

		KD	MLR	PCA	SEM, Lin (outc-free)	SEM, Non-Lin (outc-free)	SEM, Lin (outc-dep)	SEM, Non-Lin (outc-dep)
Female	(Intercept)	0.0730	21.5774	16.7431	6.2401	5.8255	-12.0379	-13.3572
		CI [-0.4763, 0.6223]	CI [20.8627, 22.2920]	CI [15.6609, 17.8254]	CI [5.7611, 6.7191]	CI [5.2811, 6.3699]	CI [-12.3512, -11.7246]	CI [-13.6813, -13.0331]
	age	-0.0014	-0.4383	-0.3401	-0.1267	-0.1228	0.1772	0.2006
		CI [-0.0122, 0.0093]	CI [-0.4523, -0.4243]	CI [-0.3613, -0.3190]	CI [-0.1361, -0.1174]	CI [-0.1334, -0.1121]	CI [0.1710, 0.1833]	CI [0.1943, 0.2070]
	Adjusted R2	-0.0002	0.4380	0.1698	0.1266	0.0952	0.3984	0.4426
	AIC	29626.8085	32172.4619	36187.6110	28301.7533	29539.6813	24195.4993	24523.2119
	BIC	29646.2606	32191.9140	36207.0632	28321.2055	29559.1334	24214.9515	24542.6640

Note: KD= Klemera-Doubal's method; MLR= multiple linear regression; PCA=principal components; SEM: Lin = linear structural equation model; SEM: Non-Lin = non-linear structural equation model. outc-dep=Outcome-dependent; outc-free=outcome-free (see sections I-III above for further details).

Appendix Table S7. Parameters of the hazard models for the outcome –dependent (OD) estimator

	SEM: Linear				SEM: Non-Linear			
	CA	95% CI	CA & FS(BA)	95% CI	CA	95% CI	CA & FS(BA)	95% CI
Females								
β	0.087	[0.09,0.08]	0.061	[0.07,0.06]	0.087	[0.09,0.08]	0.063	[0.07,0.06]
k	$5x10^{-5}$	[$3x10^{-5}$,$6x10^{-5}$]	$4x10^{-5}$	[$3x10^{-5}$,$5\ x10^{-5}$]	$5\ x10^{-5}$	[$3\ x10^{-5}$, $6\ x10^{-5}$]	$5\ x10^{-5}$	[$3\ x10^{-5}$,$7\ x10^{-5}$]
FS(BA)			1.517	[1.77,1.26]			0.014	[0.02,0.01]
Males								
β	0.080	[0.08,0.08]	0.056	[0.06,0.05]	0.080	[0.08,0.08]	0.058	[0.06,0.05]
k	$1.1x10^{-4}$	[$9x10^{-5}$,$1.5x10^{-4}$]	$1x10^{-4}$	[$7x10^{-5}$,$1.3x10^{-4}$]	$1.1x10^{-4}$	[$9x10^{-5}$,$1.5x10^{-4}$]	$8x10^{-5}$	[$6x10^{-5}$,$1.1x10^{-4}$]
FS(BA)			1.837	[2.13,1.54]			0.055	[0.06,0.05]

Note: FS(BA)= factors scores from an SEM model. Since we constrain the coefficient of CA_{t0} to be identical to the coefficient of duration, its value is the same as β in the table.

Appendix Table S8. Partial area under the curve (AUC) of the receiver operating curve (ROC) for various levels of specificity in the Gompertz survival models from Table 3 in the paper.

	KD	MLR	PCA	SEM: Linear		SEM: Non-Linear	
Parameter				Outcome-free	Outcome-dep	Outcome-free	Outcome-dep
Female: BA-CA							
80%-90%	83.72	83.25	83.78	84.09	88.82	84.02	88.43
90%-100%	71.80	71.41	71.08	72.04	80.86	72.17	80.49
0-100%	85.54	85.13	85.08	85.76	91.08	85.62	90.53
Male: BA-CA							
80%-90%	82.59	82.10	81.84	82.78	88.13	82.59	87.59
90%-100%	71.40	71.07	71.33	71.54	80.44	71.59	80.14
0-100%	84.27	83.93	83.95	84.45	90.50	84.33	89.90

Note: MLR= multiple linear regression; PCA=principal components; KD=Klemera-Doubal.

Appendix Figure S1: Representation of a simple SEM with CA and BA

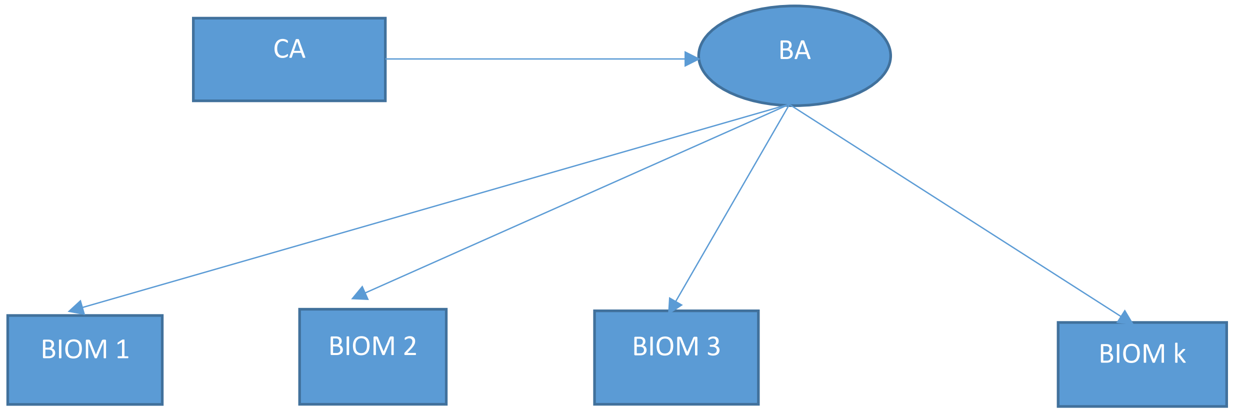

Appendix Figure S2. Age-pattern of predicted Biological Age estimated from several methods for men and women using 9 biomarkers.

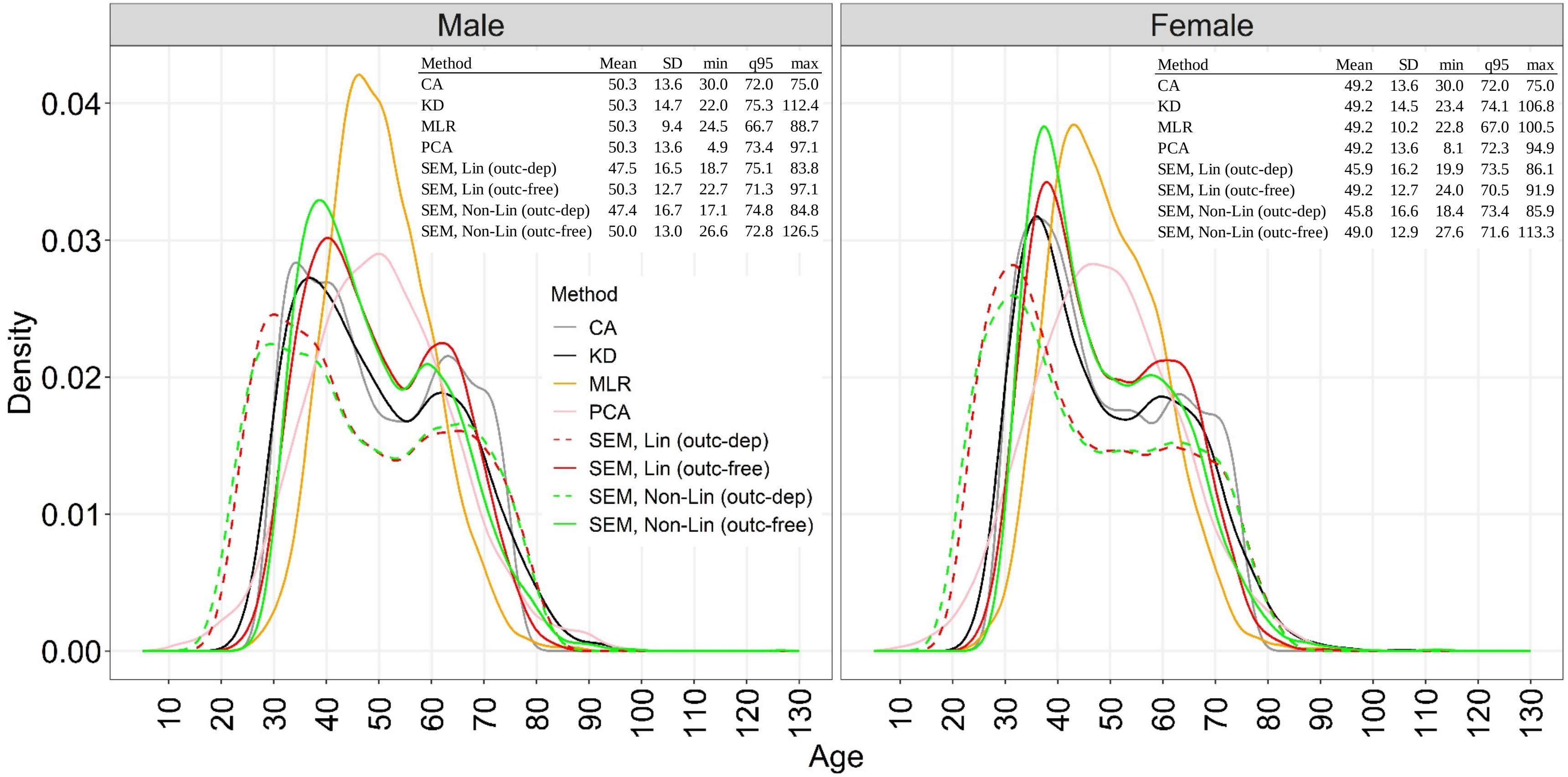

Note: CA= chronological age, PCA=principal components, MLR= multiple linear regression; KD= Klemera-Doubal's method; SEM: Lin = linear structural equation model; SEM: Non-Lin = non-linear structural equation model; SD=standard deviation; q95= 95th percentile. Outc-free: outcome-free estimator; outc-dep: outcome-dependent estimator (see methods section for further details).

Appendix Figure S3. Age-pattern of predicted Biological Age estimated from several methods for men and women in the Health and Retirement Study, 2006-2016.

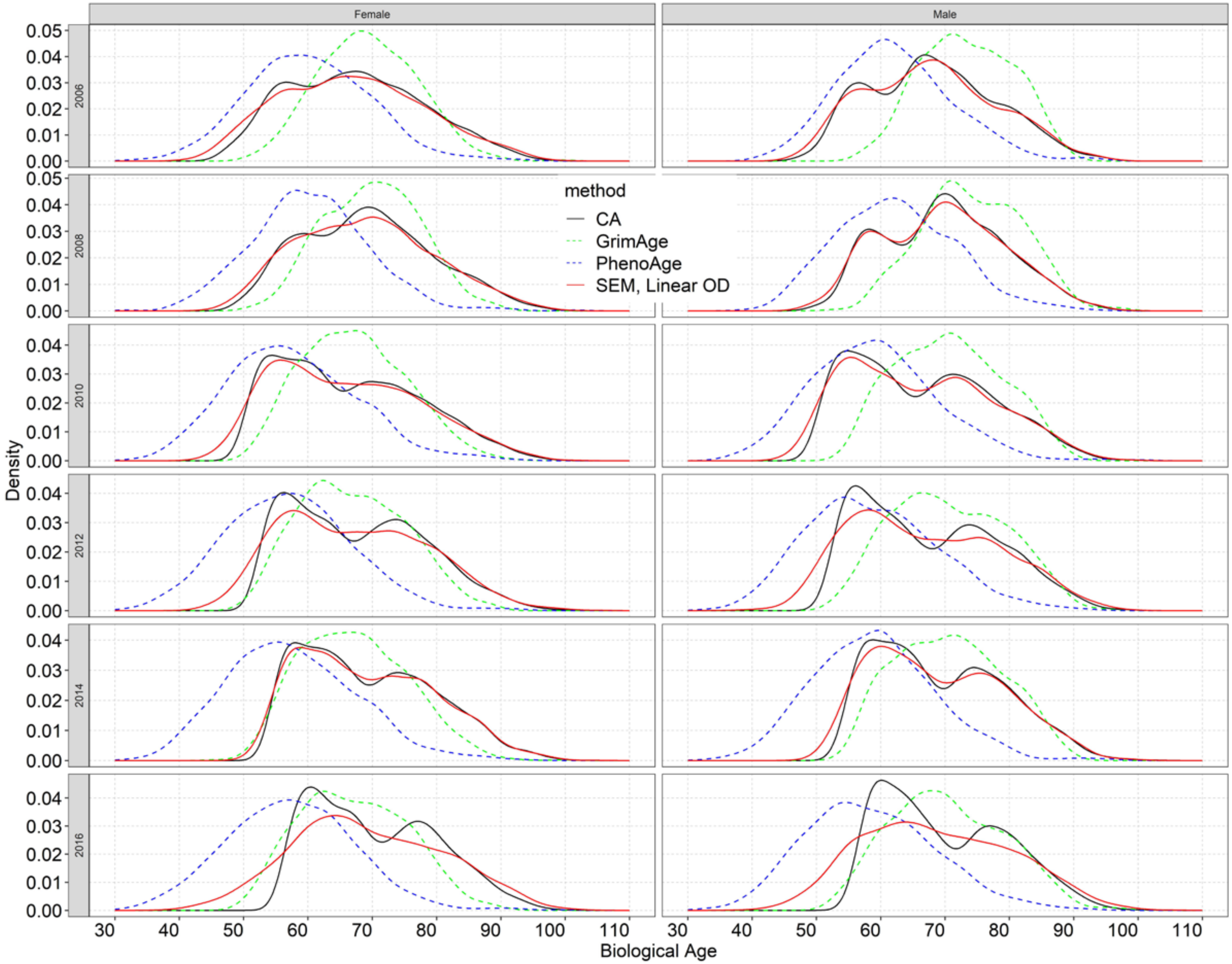

SEM linear OD (outcome-dependent) estimated with: total cholesterol, HDL cholesterol, glycosylated hemoglobin, C-reactive protein, and cystatin C. PhenoAge and GrimAge were estimated by the HRS team (see Crimmins et al. 2013, 2015, 2016, 2017, 2020).

Appendix Figure S4. Association between PhenoAge and OD, HRS 2006-2016

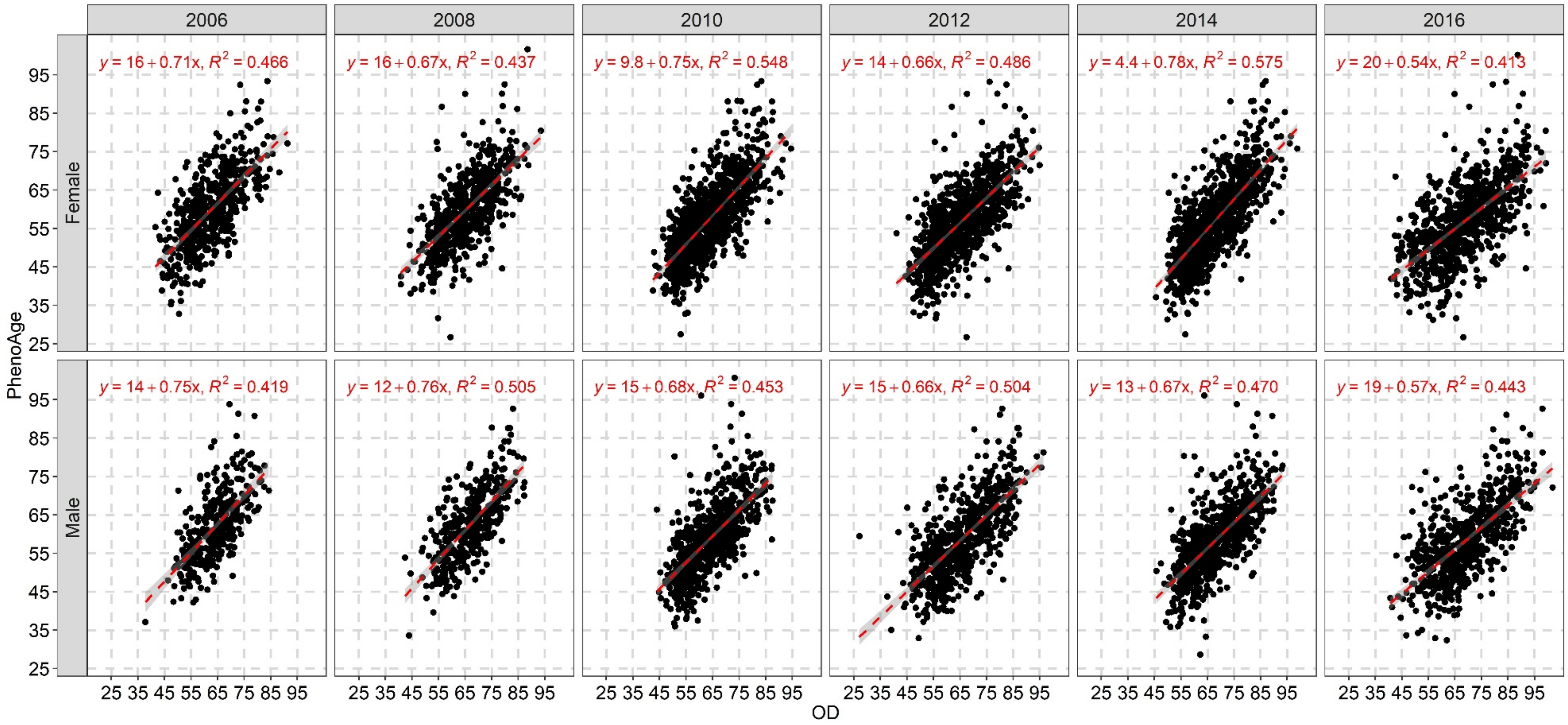

Appendix Figure S5. Association between the difference (PhenoAge-OD) and CA, HRS 2006-2016

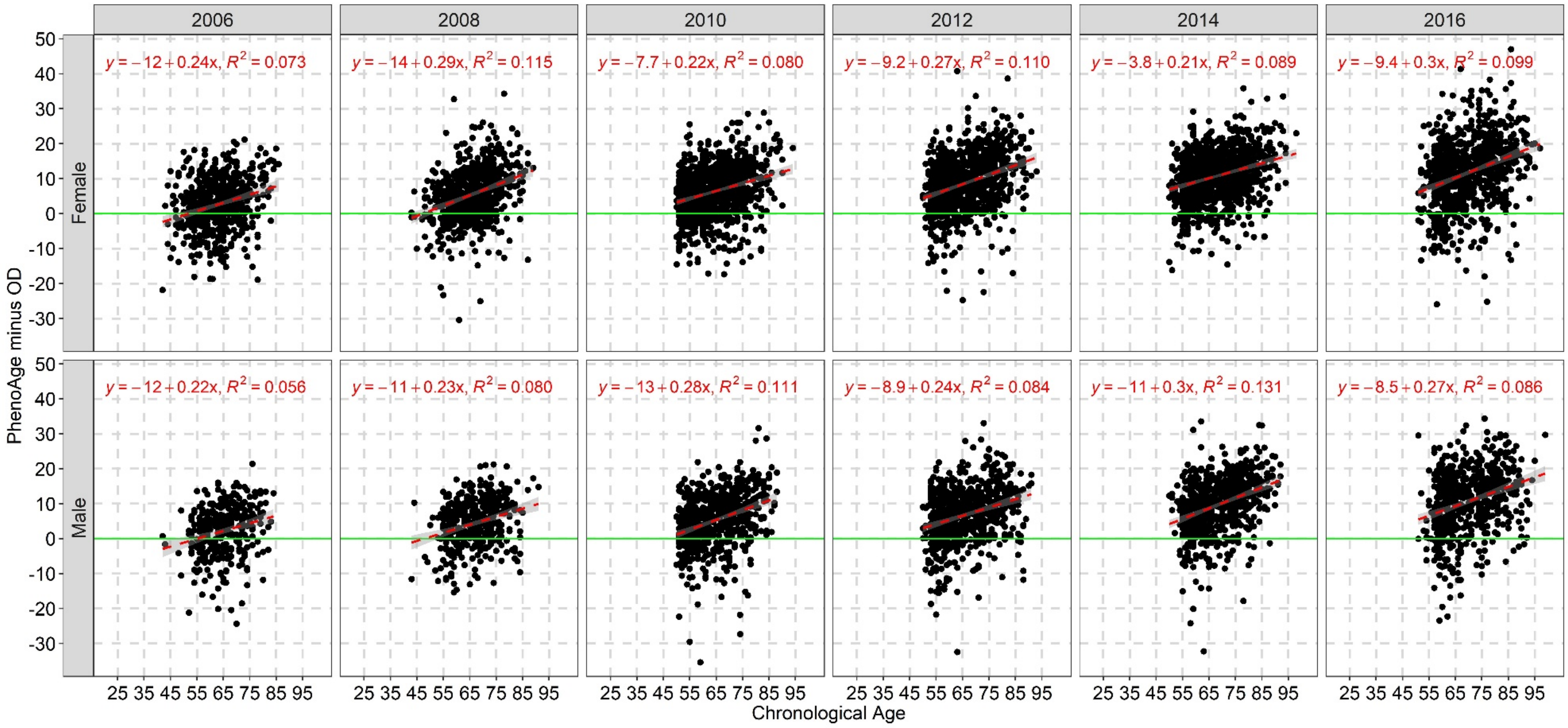

References

Institute of Medicine and National Research Council. (2013). *U.S. Health in International Perspective: Shorter Lives, Poorer Health*. https://doi.org/10.17226/13497

Crimmins, E., Faul, J., Kim, J., Weir, D., 2020. Documentation of DBS Blood-Based Biomarkers in the 2016 Health and Retirement Study. Ann Arbor MI Univ. Mich. Surv. Res. Cent.

Crimmins, E., Faul, J., Kim, J., Weir, D., 2017. Documentation of blood-based biomarkers in the 2014 Health and Retirement Study. Ann Arbor MI Univ. Mich. Surv. Res. Cent.

Crimmins, E., Faul, J., Kim, J.K., Guyer, H., Langa, K., Ofstedal, M.B., Sonnega, A., Wallace, R., Weir, D., 2013. Documentation of biomarkers in the 2006 and 2008 Health and Retirement Study. Ann Arbor MI Surv. Res. Cent. Univ. Mich.

Crimmins, E., Faul, J., Kim, J.K., Weir, D., 2015. Documentation of Biomarkers in the 2010 and 2012 Health and Retirement Study. Ann Arbor MI Surv. Res. Cent. Univ. Mich.
